# Effect of Bicarbonate of Potash on the Acidity of Urine

**Published:** 1879-01

**Authors:** 


Effect of Bicarbonate of Potash on the Ascidity of Urine.—Dr. C. H. Ralfe, teacher of Physiological Chemistry in the Medical School, St. George's Hospital, has made some observations (*Lancet*, Nov. 9, 1878) to test the effect of bicarbonate of potash on the reaction of the urine when taken before and when taken after meals.

He found that the effect of bircarbonate of potash, taken after food, on the acidity of the urine, is different from that when it is administered before meals.

For when taken on an empty stomach the acidity on the day of administration was only slightly depressed, whilst on the day following the ascidity was considerable higher than it was the day before the salt was taken. But when it was administered during the process of digestion the acidity of the urine entirely disappeared, being on two occasions neutral, and on one alkaline, whilst on the succeeding days there was no marked increase in the acidity of the urine as compared with that of the days preceding the experiment. And the same difference is observable in the hourly variations of the urine, for when the bicarbonate was taken before meals the effect of the alkali passed off at the end of two hours, and the amount of acid passed in the succeeding three hours was nearly equal to what was passed on the day no medicine was taken; whilst when the salt was taken after meals the urine remained alkaline up to the end of four hours after the dose was taken, and no recovery of acidity was noticeable.

The result of these observation tends to establish the fact that the administration of an alkaline bicarbonate on an empty stomach increases the acidity of the system, whilst its administration after a meal diminishes it.

Dr. Ralfe finds the explanation of this in that the acid reaction of the urine is generally considered due to the decomposition that occurs between an acid or an acid salt and the neutral phosphate of sodium in the blood, acid sodium phosphate being formed, which passes out with the urine. Now, one of the chief acid salts in the blood is undoubtedly bicarbonate of potash or soda, an acid salt with an alkaline reaction. It is, therefore, not surprising to find the administration of an acid salt, if it passes unaltered from the stomach into the blood, causing an increase in the acidity of the urine. And this is, indeed, what happens when a dose of bicarbonate of potash and soda is taken into the stomach before meals, for then the mucous membrane under normal conditions being either neutral or alkaline, the bicarbonate is absorbed undecomposed into the blood, and causes the increase in acidity of the urine which has been noted. On the other hand, when the salt is taken during the digestion, the acid contents of the stomach decompose it, carbonic acid is liberated, which escapes by the mouth, whilst the alkaline bases pass into the system and cause the urine to assume an alkaline reaction.

The therapeutic indications to be drawn from these observations may be thus summarized:—

1. In cases of acid dyspepsia arising from the excessive formation of acid within the system, as in lithæmia, the alkaline bicarbonates should not be administered before food, but after.

2. The administration of alkaline bicarbonates before meals is indicated in those cases where the free acid is formed in the stomach itself, the result of fermentative changes of undigested food or morbid mucus, when it is necessary to diminish the too high degree of acidity thus caused in order to permit digestion to be properly performed.—*American Journal of Medical Sciences.*

